# Nrf2-Activating Bioactive Peptides Exert Anti-Inflammatory Activity through Inhibition of the NF-κB Pathway

**DOI:** 10.3390/ijms23084382

**Published:** 2022-04-15

**Authors:** Federica Tonolo, Alessandra Folda, Valeria Scalcon, Oriano Marin, Alberto Bindoli, Maria Pia Rigobello

**Affiliations:** 1 Department of Biomedical Sciences, University of Padova, Via Ugo Bassi 58/b, 35131 Padova, Italy; federica.tonolo@unipd.it (F.T.); alessandra.folda.1@unipd.it (A.F.); valeria.scalcon@unipd.it (V.S.); oriano.marin@unipd.it (O.M.); 2 Institute of Neuroscience, CNR, Via G Colombo 3, 35131 Padova, Italy; alberto.bindoli@bio.unipd.it

**Keywords:** bioactive peptides, antioxidant, anti-inflammatory, Nrf2, NF-κB

## Abstract

Redox status and inflammation are related to the pathogenesis of the majority of diseases. Therefore, understanding the role of specific food-derived molecules in the regulation of their specific pathways is a relevant issue. Our previous studies indicated that **K-8-K** and **S-10-S**, milk and soy-derived bioactive peptides, respectively, exert antioxidant effects through activation of the Keap1/Nrf2 pathway. A crosstalk between Nrf2 and NF-κB, mediated by the action of heme oxygenase (HO-1), is well known. On this basis, we studied if these peptides, in addition to their antioxidant activity, could exert anti-inflammatory effects in human cells. First, we observed an increase of HO-1 expression in Caco-2 cells treated with **K-8-K** and **S-10-S**, following the activation of the Keap1/Nrf2 pathway. Moreover, when cells are treated with the two peptides and stimulated by TNF-α, the levels of NF-κB in the nucleus decreased in comparison with TNF-α alone. In the same conditions, we observed the downregulation of the gene expression of proinflammatory cytokines (*IL1B*, *IL6*, and *TNF*), while the anti-inflammatory cytokine gene, *IL1RN*, was upregulated in Caco-2 cells processed as reported above. Then, when the cells were pretreated with the two peptides and stimulated with LPS, a different proinflammatory factor, (TNF-α) was estimated to have a lower secretion in the supernatant of cells. In conclusion, these observations confirmed that Nrf2-activating bioactive peptides, **K-8-K** and **S-10-S**, exerted anti-inflammatory effects by inhibiting the NF-κB pathway.

## 1. Introduction

Considering the constant increase in lifespan of the world population, chronic and age-related diseases, such as diabetes, cardiovascular diseases, and obesity characterized by inflammatory processes associated with an imbalance of cellular redox status, are gaining increasing relevance. For this reason, scientists and industries are focusing their attention on new strategies to cope with these diseases. A new approach regards nutraceuticals, which are food-derived bioactive molecules that promote health benefits beyond basic nutritional values. Among these compounds, bioactive peptides, polyphenols, and other specific constituents are gaining great interest [1]. Food-derived bioactive peptides are fragments of proteins that are inactive in the native structure of the macromolecules and show health-promoting effects on many body functions, e.g., antihypertensive, opioid agonist or antagonist, antimicrobial, and antioxidant properties [2,3]. These peptides can be released from food proteins through different mechanisms, such as gastrointestinal digestion, food processing, fermentation, and enzymatic hydrolysis [4]. Up to now, their mechanism of action has been scarcely explored and, in most cases, is still unknown.

**Figure 1:**
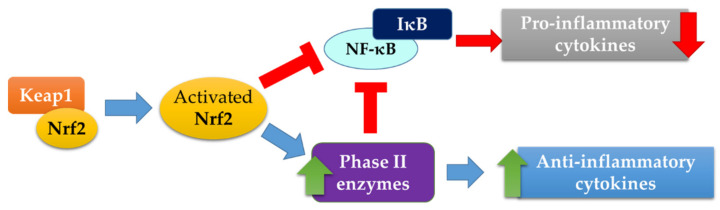
Schematic representation of the crosstalk between antioxidant and proinflammatory pathways. Keap1: Kelch-like ECH-associated protein 1; Nrf2: nuclear factor erythroid 2-related factor 2; NF-κB: nuclear factor kappa-light-chain-enhancer of activated B cells; IκB: inhibitor of nuclear factor kappa B.

We previously observed that some milk and soy bioactive peptides exert their antioxidant action through the activation of the Keap1/Nrf2 pathway, the main pathway involved in the regulation of redox homeostasis [5,6,7,8]. Some of the studied peptides act as disruptors of the interaction between Keap1 and Nrf2, promoting the translocation of the transcription factor from the cytosol to the nucleus, where it can bind to the antioxidant response element (ARE) sequences, inducing the transcription of antioxidant and phase II genes, such as thioredoxin reductase (TrxR), glutathione reductase (GR), and NADPH quinone oxidoreductase 1 (NQO1). These results give the opportunity to gain new insights into the mechanism of Keap1/Nrf2 pathway activation. Indeed, oxidants and electrophiles cause transient modifications of Keap1, by interacting with specific Cys residues, activating this signaling pathway. As reported above, in our previous research, we observed that some antioxidant bioactive peptides can interact directly with the amino-acid residues of the pocket of Keap1 involved in the binding of the Neh domain of Nrf2, inhibiting the interaction of these two proteins [5,6,7]. It is well known that Nrf2 regulates, among other functions, the stability and the expression of many transcriptional regulators [9]. In addition, Nrf2 cooperates with nuclear factor kappa-light-chain-enhancer of activated B cells (NF-κB) in order to maintain redox and inflammatory homeostasis through a highly regulated crosstalk (Figure 1) [10,11,12]. NF-κB is an inducible transcription factor, and its activation involves two different pathways. In the canonical system, NF-κB activation is mediated in an IKK-dependent manner. Indeed, the inhibitory protein IκB (inhibitor of nuclear factor kappa B) is phosphorylated by IKK (IκB kinase) and subsequently eliminated by the proteasome. This process promotes the migration of NF-κB from the cytosol to the nucleus, leading to the transcription of the downstream regulated genes. The main function of NF-κB is the regulation of the inflammatory response, inducing the expression of various proinflammatory genes, such as interleukin-1 beta (IL-1β), IL-6, and tumor necrosis factor-alpha (TNF-α), but it can also regulate cell proliferation, apoptosis, morphogenesis, and differentiation [13]. Nrf2 negatively regulates the NF-κB signaling pathway through various mechanisms. The activation of the Keap1/Nrf2 system causes a decrease in the intracellular ROS levels, and, as it is known that NF-κB is triggered by oxidative stress, its action is, therefore, inhibited by activated Nrf2. Moreover, the latter inhibits the translocation of NF-κB to the nucleus, directly preventing IκB degradation, as well as through the action of heme oxygenase-1 (Nrf2/HO-1 axis) and other phase II enzymes (Figure 1) [10,14,15].

In this study, two antioxidant bioactive peptides, **K-8-K** (KVLPVPEK) and **S-10-S** (SLVNNDDRDS), derived from milk and soy, respectively, were taken into account as they demonstrated a high capacity of Nrf2 stimulation [5,7]. Knowing the crosstalk between Nrf2 and NF-κB, the aim of this work was to analyze if these food-derived peptides, in addition to their antioxidant activity, can exert anti-inflammatory effects in a cell model.

## 2. Results

### 2.1. HO-1 Expression Increases in Cells Treated with K-8-K and S-10-S

**Figure 2:**
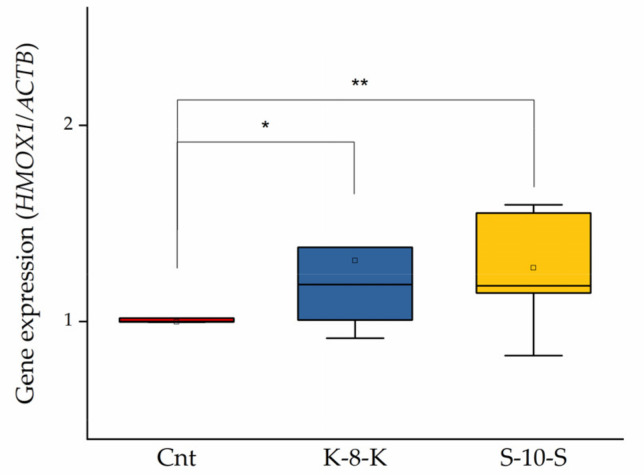
mRNA expression analysis of *HMOX1*. Caco-2 cells were treated with 0.05 mg/mL **K-8-K** and **S-10-S** for 24 h, and then mRNA was purified as described in Section 4. qRT-PCR analysis of *HMOX1* expression was performed to estimate its potential overexpression. β-Actin (*ACTB*) was used as a reference. Means of at least three replicates estimated in duplicate were compared (** *p* < 0.01, * *p* < 0.05). Cnt: untreated cells.

As reported above, the activation of Keap1/Nrf2 leads to overexpression of antioxidant and phase II enzymes. In particular, in our previous studies, we found an increased activity of some enzymes (TrxR, GR, SOD1 and NQO1) by Nrf2 signaling activation in cells treated with **K-8-K** and **S-10-S [5,7].** Moreover, we studied HO-1 as it is related to the inhibition of NF-κB pathway [16]. Therefore, gene expression of this protein in Caco-2 cells treated for 24 h in the presence of **K-8-K** and **S-10-S** was evaluated. With the peptides, the expression of *HMOX1* significantly increased to 30% of the untreated control (Cnt) (Figure 2).

### 2.2. Changes in Nuclear NF-κB and Cytosolic IκB Levels in Cells Treated with the Antioxidant Peptides

**Figure 3:**
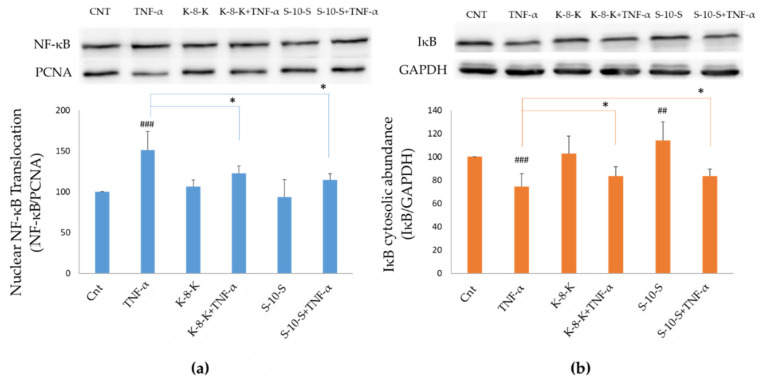
Evaluation of the levels of NF-κB and IκB. Caco-2 cells were preincubated with 0.05 mg/mL peptides for 24 h, and the inflammatory stimulus was induced with 2 ng/mL TNF-α for 2.5 h. Western blot analysis of the abundance of NF-κB in nuclear fractions (**a**) and cytosolic IκB (**b**) was performed. Densitometric estimation of the reported WB was carried out using PCNA or GAPDH as the loading control for the nuclear and cytosolic compartments, respectively. Means of at least three experiments were compared. ### *p* < 0.001, ## *p* < 0.01 vs. nontreated group (Cnt); * *p* < 0.05 vs. only TNF-α-treated group.

Following the observation that the expression of HO-1 was increased in the presence of **K-8-K** and **S-10-S**, the activation of NF-κB was analyzed in the same cells treated with the two peptides for 24 h followed by inflammation induction with 2 ng/mL TNF-α for 2.5 h. When activated, NF-κB translocates from the cytosol to the nucleus as its inhibitor IκB is phosphorylated and degraded by the proteasome. Thus, NF-κB nuclear levels were estimated after separation of cytosolic and nuclear fractions. As reported in Figure 3a, after TNF-α stimulus, NF-κB nuclear translocation increased of the 50% compared to the negative control. On the other hand, in the cells pretreated with **K-8-K** and **S-10-S** followed by TNF-α stimulus, there was a remarkable reduction in NF-κB nuclear translocation of 28% and 36%, respectively, in comparison to the positive control. Moreover, as apparent from Figure 3b, in cells treated with the peptides, IκB cytosolic levels increased when compared to the control. In the cells subjected to TNF-α stimulus, but in the presence of **K-8-K** and **S-10-S**, IκB increased by 14% and almost 10%, respectively. In fact, Nrf2 activation leads to the inhibition of NF-κB, consequent to an increase in IκB concentration in the cytosol. Of note, **S-10-S** exhibited a slightly greater inhibition of the NF-κB pathway than **K-8-K**.

### 2.3. Differences in Anti- and Proinflammatory Cytokine Levels

#### 2.3.1. TNF-α, IL-1β, and IL-6 Gene Expression Decreases in the Presence of the Two Peptides

**Figure 4:**
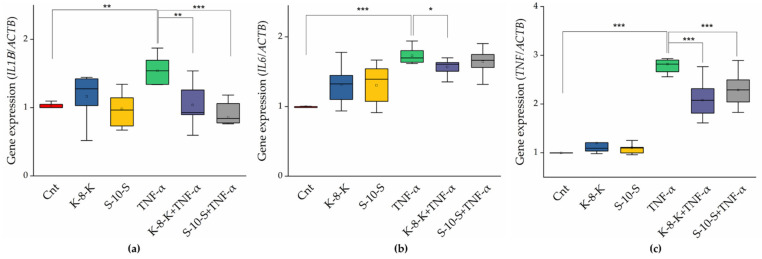
Gene expression of cytokines regulated by the NF-κB pathway in Caco-2 cells treated with **K-8-K** and **S-10-S**. qRT-PCR analysis of proinflammatory cytokines, IL-1β (*IL1B*, (**a**)), IL-6 (*IL6*, (**b**)), and TNF-α (*TNF,* (**c**)). Gene expression was carried out in mRNA samples of Caco-2 cells treated with **K-8-K** and **S-10-S**. Inflammation was induced by 2 ng/mL TNF-α. β-Actin (*ACTB*) was used as a reference. Means of at least three replicates estimated in duplicate were compared (*** *p* < 0.001, ** *p* < 0.01, * *p* < 0.05). Cnt: untreated cells.

The gene expression of NF-κB-regulated cytokines (IL-1β, IL-6, and TNF-α) in the presence of **K-8-K** and **S-10-S** was examined by qRT-PCR. *IL1B*, *IL6*, and *TNF* expression increased in the presence of the inflammatory stimulus elicited by TNF-α, but it was significantly lower in cells pretreated with the two peptides and then stimulated with TNF-α (Figure 4a–c, respectively). This observation confirmed the decreased activity of this transcription factor. In particular, **S-10-S** exhibited a high capacity of inhibiting the expression of IL-1β, which resulted in a decrease of up to 20% with respect to the positive control.

#### 2.3.2. Secreted TNF-α Levels Decrease after Pretreatment of Caco-2 Cells with Peptides

**Figure 5:**
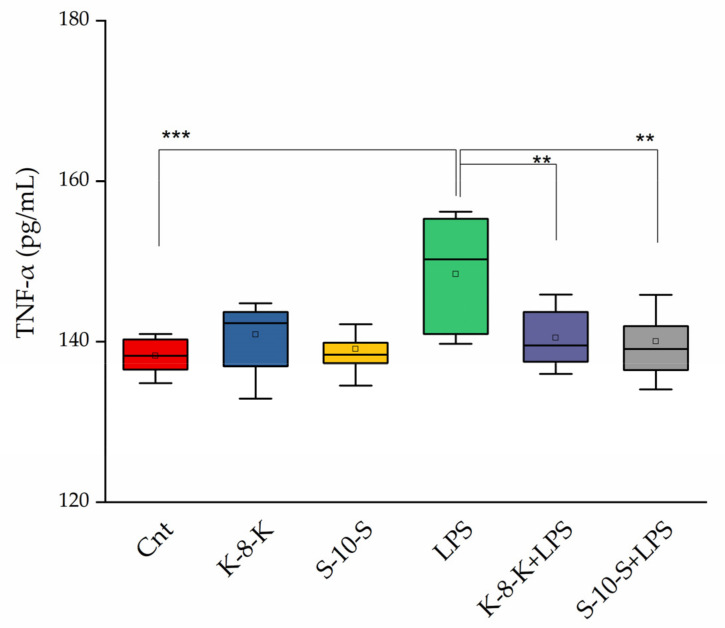
Effect of **K-8-K** and **S-10-S** on the inflammatory response in Caco-2 cells treated with 0.01 mg/mL LPS. The levels of TNF-α were measured by ELISA assay in supernatants of Caco-2 cells grown for 14 days and pretreated with the peptides (0.05 mg/mL) for 24 h. Here, 0.01 mg/mL LPS was used as inflammatory stimulus. Means of at least three replicates estimated in duplicate were compared (*** *p* < 0.001, ** *p* < 0.01). Cnt: untreated cells.

To further confirm the decreased activity of the NF-κB pathway, secreted TNF-α levels were estimated using an ELISA assay. To this purpose, another proinflammatory stimulus, LPS, was employed, to avoid the direct addition of TNF-α to the cells which could blur the estimations. Caco-2 cells were grown for 14 days and pretreated with the peptides; then, the inflammatory condition was induced by using 0.01 mg/mL LPS. As evident from Figure 5, TNF-α levels in cells pretreated with **K-8-K** and **S-10-S** were significantly lower in comparison with the positive control, treated only with LPS.

#### 2.3.3. Anti-Inflammatory Cytokine, IL-1ra, Increases in the Presence of the Peptides

**Figure 6:**
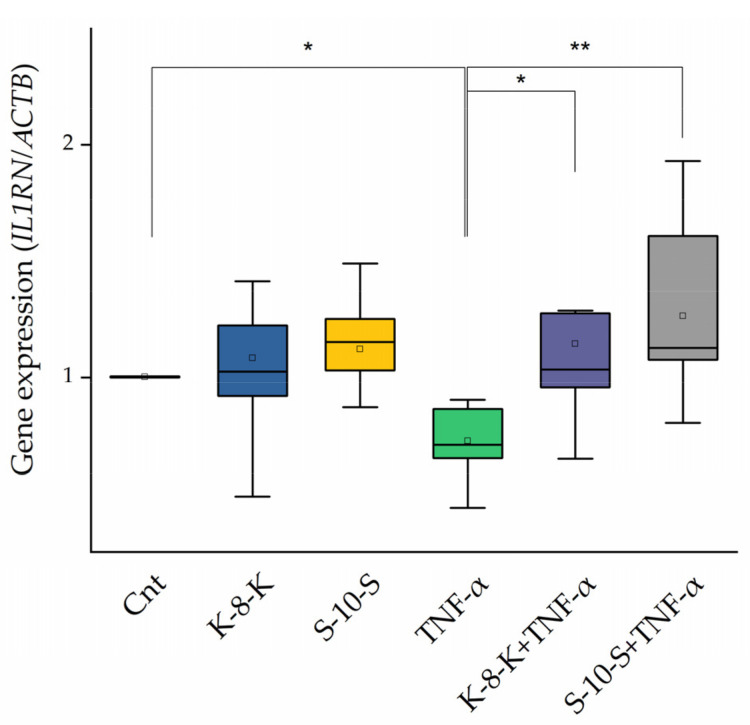
Gene expression analysis of anti-inflammatory cytokines in Caco-2 cells treated with **K-8-K** and **S-10-S**. qRT-PCR analysis of anti-inflammatory cytokine, IL-1ra, gene expression (*IL1RN*) was carried out in mRNA samples of Caco-2 cells treated with **K-8-K** and **S-10-S** subjected to induction of inflammation by 2 ng/mL TNF-α. β-Actin (*ACTB*) was used as a reference. Means of at least three replicates estimated in duplicate were compared (** *p* < 0.01, * *p* < 0.05). Cnt: untreated cells.

The expression of an anti-inflammatory cytokine, IL-1ra, was also evaluated. In Caco-2 cells pretreated with **K-8-K** and **S-10-S,** inflammation was induced by 2 ng/mL TNF-α. As reported in Figure 6, when the cells were pretreated with the two peptides, *IL1RN* expression was found close to basal control levels compared to positive control treated only with TNF-α.

## 3. Discussion

Considering that imbalance of the redox status and inflammation are related to the pathogenesis of many diseases, the knowledge of the molecular role of specific food-derived molecules in the regulation of specific pathways appears relevant. NF-κB and Keap1/Nrf2 systems are the two major signaling pathways operating in these processes. The transcription factors Nrf2 and NF-κB are redox-sensitive molecular switches acting on a large range of biochemical functions related to oxidative stress and immunomodulation [12]. It is well known that NF-κB stimulates and maintains an inflammatory condition by inducing the expression of a large range of proinflammatory factors such as cytokines, chemokines, adhesion molecules, and enzymes mediating inflammation such as cyclooxygenase-2 (COX-2) and the inducible NO synthase (iNOS). On the contrary, the transcription factor Nrf2 appears to contrast inflammation by expressing enzymes removing hydrogen peroxide and other oxidants or preventing oxidative stress in other ways, such as by heme oxygenase [10,16]. Hence, the equilibrium of the two pathways appears critical for maintaining a healthy cell. As both Keap1/Nrf2 and NF-κB complexes contain redox-sensitive cysteines, the enzymatic thiol redox systems depending on glutathione and thioredoxin modulate the balance involving the proinflammatory action of NF-κB and the antioxidant signaling pathway mediated by Nrf2 [12]. A crosstalk involving both complexes has, therefore, been envisaged, as several molecules decrease NF-κB activation upon stimulation of Nrf2 [16,17,18]. In the relationship involving Nrf2 and NF-κB, a relevant role is played by the Nrf2-induced heme oxygenase-1 (HO-1) exhibiting an adaptive response where either the link between Nrf2 and NF-κB or the related antioxidant and anti-inflammatory pathways are operative [16,17].

In our diet, some molecules, such as glucosinolates, exert anti-inflammatory effects through the activation of the Nrf2 pathway [19,20,21]. On the other hand, many reviews and papers ascribed the anti-inflammatory properties to bioactive peptides as they prevent the production of NO and proinflammatory cytokines (TNF- α, IL-6, and IL-1β) [22,23].

Our previous studies regarding the analysis of bioactive peptides derived from fermented milk or soy showed that some peptides can exert antioxidant effects through the activation of the Keap1/Nrf2 pathway [5,6,7]. In particular, **K-8-K** and **S-10-S**, derived from milk and soy, respectively, were the most active peptides in inducing this antioxidant pathway. Indeed, they increased the nuclear translocation of Nrf2 and, consequently, the expression of ARE-controlled genes of antioxidant and phase II enzymes such as NQO1, TrxR, and GR [5,7].

Considering that activated Nrf2 regulates NF-κB, we investigated if these peptides, in addition to their antioxidant activity, could exert anti-inflammatory effects in human cells. Generally, Nrf2 inhibits NF-κB translocation from the cytosol to the nucleus by preventing IκB degradation. Relevant to the anti-inflammatory effect is the overexpression of HO-1, which further upregulates anti-inflammatory cytokines and affects the NF-κB pathway activation [14,19,20,24].

On this basis, HO-1 gene expression was analyzed in cells pretreated with the two antioxidant peptides, and we observed that *HMOX1* expression was increased, confirming the activation of the Keap1/Nrf2 pathway and the possible action of Nrf2/HO-1 axis.

Moreover, the effects of Nrf2 and HO-1 activation induced by the presence of **K-8-K** and **S-10-S** were evaluated in cells stimulated with TNF-α, and the translocation of NF-κB from the cytosol to the nucleus and the cytosolic IκB abundance were followed. In cells pretreated with the two peptides followed by TNF-α-induced inflammation, we observed that IκB levels increased in the cytosol with concomitantly decreased levels of NF-κB in the nucleus. These results could be explained by the direct action of Nrf2 on the inhibition of IκB degradation, due to the high Nrf2 activation in cells treated with the two peptides. Of note, **S-10-S** increased IκB levels in non inflammatory conditions, suggesting that this peptide has also an anti-inflammatory effect in physiological conditions. Moreover, inhibition of the NF-κB pathway leads to a decrease in the expression of the proinflammatory cytokines IL-1β, IL-6, and TNF-α. To this purpose, the expression of *IL1B*, *IL6*, and *TNF* was estimated in cells pretreated with **K-8-K** and **S-10-S,** and then inflammation was induced by TNF-α. It was shown that the expression of proinflammatory cytokines was downregulated in the studied conditions. In order to confirm the obtained results, secreted TNF-α was estimated through ELISA assay in the supernatant of Caco-2 cells treated with another proinflammatory stimulus, LPS. Of note, the level of this cytokine, with respect to the positive control, was lower in the cells pretreated with **K-8-K** and **S-10-S**.

As reported in Section 1, the overexpression of HO-1 leads to an increase in anti-inflammatory cytokine production. Consequently, the expression of IL-1ra, the inhibitor of interleukin-1 receptor (IL-1R), was studied. As a result, *IL1RN* expression increased in cells treated with the peptides and TNF-α.

**Figure 7:**
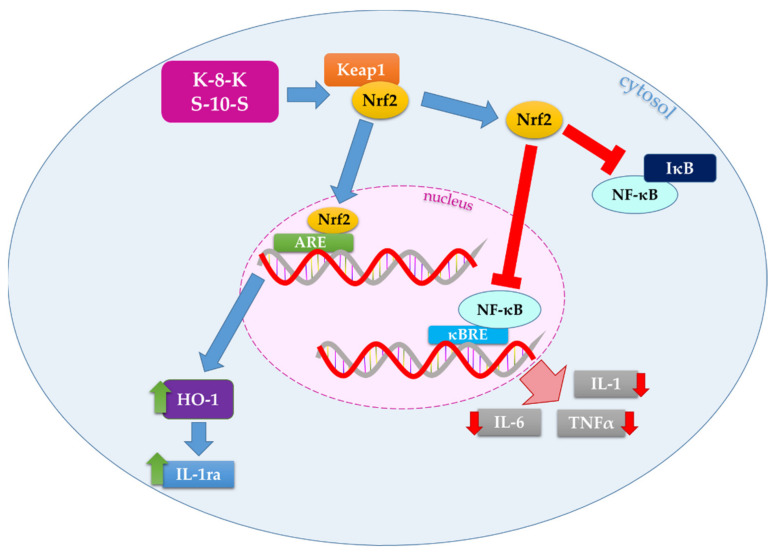
Schematic representation of the molecular mechanism of the antioxidant bioactive peptides, exerting anti-inflammatory effects by inhibiting the NF-κB pathway. Keap1: Kelch-like ECH-associated protein 1; Nrf2: nuclear factor erythroid 2-related factor 2; NF-κB: nuclear factor kappa-light-chain-enhancer of activated B cells; IκB: inhibitor of nuclear factor kappa B; ARE: antioxidant response element; κBRE: κB response element; HO-1: heme oxygenase-1.

In conclusion, our observations suggest that Nrf2-activating bioactive peptides, **K-8-K** and especially **S-10-S**, can exert anti-inflammatory effects by inhibiting the NF-κB pathway through both the direct action of the activated Nrf2 and the effects of the phase II enzymes regulated by this transcription factor, particularly HO-1, highlighting potentially useful implications for human health (Figure 7).

## 4. Materials and Methods

### 4.1. Bioactive Peptides Synthesis

**K-8-K** and **S-10-S** were synthesized by a solid-phase technique using a fully automated peptide synthesizer (Syro II, MultiSynTech Gmbh, Witten, Germany), as previously reported [5,7,25].

### 4.2. Caco-2 Cell Line

Caco-2 cells, kindly provided by DISCOG (University of Padova, Padova, Italy), were used as a model in this research. This cell line is derived from colorectal adenocarcinoma. Caco-2 cells were cultured in DMEM supplemented with 10% FBS, 10,000 units/mL of penicillin, and 1 mg/mL of streptomycin, at 37 °C in a humidified atmosphere containing 5% CO_2_. The cells used in this study were between 35 and 60 passages.

### 4.3. Analysis of NF-κB Pathway

#### 4.3.1. Isolation of Subcellular Fractions

In order to evaluate the translocation of NF-κB to the nucleus, nuclear and cytosolic fractions were separated by following the procedure previously described [5,6,7]. Cells (1 × 10^6^) were seeded in T25 flasks, grown for 48 h in complete medium, and then treated with **K-8-K** and **S-10-S** (0.05 mg/mL) for 24 h. To induce inflammatory condition, the cells were treated with 2 ng/mL TNF-α for 2.5 h before harvesting them. Then, cells were washed with 1 mL of PBS 1× and lysed with 100 µL of buffer containing 10 mM Hepes/Tris pH 7.9, 10 mM KCl, 0.1 mM EDTA, 0.1 mM EGTA, 0.1 mM PMSF, 1 mM NaF, and a protease inhibitor cocktail (Complete, Roche^®®^) for 15 min, at 4 °C. Afterward, 20 µL of IGEPAL (5%) was added to the samples, before mixing vigorously for 15 s. Subsequently, cell lysates were centrifuged at 1000× *g* for 10 min at 4 °C; the pellet constituted the nuclear fraction, while the supernatant was the crude cytosolic fraction. The pellets were dissolved in 20 mM Hepes/Tris (pH 7.9), 0.4 M NaCl, 1 mM EDTA, 1 mM EGTA, in the presence of 0.1 mM PMSF, 1 mM NaF, and a protease inhibitor cocktail. Samples were kept on ice for 15 min, vigorously mixed every 2 min for 10–15 s, and then centrifuged at 20,000× *g* for 10 min at 4 °C to discard the debris. Proteins were estimated according to Lowry et al. (1951) [26].

#### 4.3.2. Preparation of Caco-2 Cells Lysates

Cells were seeded and treated as reported in Section 4.3.1, and then harvested, washed with 1 mL of PBS 1×, and lysed with 150 µL of lysis buffer (RIPA buffer modified), containing 1% Triton X 100, 150 mM NaCl, 0.1%, SDS, 1 mM NaF, 0.5% DOC, 1 mM EDTA, 5 mM Tris/HCl (pH 7.4), 0.1 mM PMSF, and a protease inhibitor cocktail. Subsequently, samples were incubated at 4 °C for 45 min, vigorously mixed every 5 min, and aspirated with a 22-gauge needle every 10 min; at the end of the incubation, cell lysates were centrifuged for 5 min at 11,600× *g* in order to discard debris. Proteins were determined according to Lowry et al. (1951) [26].

#### 4.3.3. Western Blot Analysis

The expression level of NF-κB and IκB was determined by Western blot (WB). Samples of 30 µg of proteins were subjected to SDS-PAGE (10%) and transferred onto a nitrocellulose membrane (Santa Cruz Biotechnology Inc., Santa Cruz, CA, USA) using Trans Blot^®®^ Turbo™ (BIORAD, Hercules, CA, USA), before probing with the primary monoclonal antibodies anti-NF-κB (Santa Cruz Biotechnology Inc., Santa Cruz, CA, USA) and anti-IκB (Cell Signaling Technology, MA, USA). The WB detection was obtained using an ECL system with UVITEC (Alliance Q9 Advanced, Cambridge, UK) equipment. Densitometric analysis of WB bands was performed using NineAlliance software. As loading references, PCNA and GAPDH (Santa Cruz Biotechnology Inc., Santa Cruz, CA, USA) were employed.

#### 4.3.4. Gene Expression Analysis of Cytokines in Caco-2 Cells by qRT-PCR

The levels of gene expression of *HMOX1* and various cytokines (*IL1RN*, *IL1B*, *IL6*, and *TNF*) were evaluated with qRT-PCR, and β-actin was used as a reference. Caco-2 cells (5 × 10^5^) were seeded into six-well plates and, after 48 h, treated with the peptides (0.05 mg/mL) for 24 h. To determine the inflammation process, cells were treated with 2 ng/mL TNF-α for 2.5 h. After treatment, cells were harvested, rinsed with 1 mL of PBS 1×, and lysed with 1 mL of TRIzol reagent (Invitrogen, Thermo Fisher Scientific, Waltham, MA, USA). mRNA was extracted with chloroform and then precipitated with isopropanol. Samples were rinsed, and, after centrifugation at 7500× *g*, the pellets were air-dried. The extracted mRNA was diluted in 10 µL of RNase-free water, and the concentration was measured using NanoDrop system (Thermo Fisher Scientific, Waltham, MA, USA). mRNA (1 µg) was subjected to reverse transcription using LunaScript™ RT SuperMix Kit (New England Biolabs, Ipswich, MA, USA). The resulting cDNA (1.5 ng/µL) was used for the qRT-PCR analysis utilizing Luna^®®^ Universal qPCR Master Mix (New England Biolabs, Ipswich, MA, USA). The target cDNA was amplified as follows: an initial step of denaturation at 95 °C for 1 min, and then 42 cycles of denaturation for 15 s at 95 °C and extension at 60 °C for 30 s. The sequences of the used primers are indicated in Table 1.

#### 4.3.5. Evaluation of Secreted TNF-α Levels Using ELISA Kit

Caco-2 cells (2 × 10^5^) were seeded into six-well plates and grown in complete medium for 14 days. Then, the medium was replaced by DMEM/F12, and the cells were treated with the two peptides (0.05 mg/mL) for 24 h. Inflammation stress was induced using 0.01 mg/mL LPS. The TNF-α concentration in cell supernatants was determined employing a human TNF-α ELISA kit according to the instructions of the manufacturer (Diaclone SAS, Besancon Cedex, France). Briefly, 100 μL of cell supernatant and TNF-α standard were added in duplicate to the wells. Samples were incubated for 3 h at room temperature with biotinylated anti-TNF-α at room temperature. The plate was washed three times with 300 µL of washing buffer. Then, 100 μL of streptavidin–HRP was added into the wells, and the plate was incubated for 30 min at room temperature and then washed three times. At this point, 100 µL of ready-to-use 3,3′,5,5′-tetramethylbenzidine substrate solution was added to each well and incubated for 15 min at room temperature. At the end, 100 µL of H_2_SO_4_ (stop reagent) was added, and the absorbance was detected at 450 nm using a plate reader (TECAN Infinite^®®^ M200 PRO, Männedorf, Switzerland).

### 4.4. Statistical Analysis

Values are indicated as the mean ± SD of at least three independent experiments; the replicates were analyzed at least in duplicate. ANOVA was performed using the Tukey–Kramer multiple comparison test through OriginPro software (OriginLab Corporation, Northampton, MA, USA).

## 5. Conclusions

In conclusion, the obtained results showed that the antioxidant bioactive peptides, **K-8-K** and **S-10-S,** which activate the Keap1/Nrf2 signaling pathway, exert anti-inflammatory effects through inhibition of the NF-κB pathway, shedding light on the knowledge of the molecular mechanism of bioactive peptides. Knowing that chronic and age-related diseases, such as diabetes, cardiovascular disease, and obesity are characterized by an alteration of inflammatory processes and an imbalance of cellular redox status, the advances originating from this research appear of interest as these molecules exert both antioxidant and anti-inflammatory effects. In particular, these bioactive peptides could be used as new strategies to address the need of reducing the risk of occurrence of the above-reported diseases.

## Figures and Tables

**Table 1:** Sequences of the primers used for qRT-PCR.

Protein	Gene	Forward	Reverse
HO-1	*HMOX1*	5′–CCA GCG GGC CAG CAA CAA AGT GC–3′	5′–AAG CCT TCA GTG CCC ACG GTA AGG–3′
IL-1ra	*IL1RN*	5′–GAA GAT GTG CCT GTC CTG TGT–3′	5′–CGC TCA GGT CAG TGA TGT TAA–3′
IL-1β	*IL1B*	5′ –GGA CAG GAT ATG GAG CAA CA–3′	5′–GGC AGA CTC AAA TTC CAG CT–3′
IL-6	*IL6*	5′–ACC TGA ACC TTC CAA AGA TGG C–3′	5′–TCA CCA GGC AAG TCT CCT CAT TG–3′
TNF-α	*TNF*	5′–CCC AGG CAG TCA GAT CAT CTT CTC GGA A–3′	5′–CTG GTT ATC TCT CAG CTC CAC GCC ATT–3′
β-actin	*ACTB*	5′–ACCTGACTGACTACCTCATGAAGA–3′	5′–GCGACGTAGCACAGCTTCTC–3′

## Data Availability

Not applicable.
